# Antimicrobial changes made for suspected infectious diarrhea after positive gastrointestinal pathogen panel result

**DOI:** 10.1017/ash.2025.10184

**Published:** 2025-10-20

**Authors:** Natalie A. Mackow, Madison G. Ponder, Samantha R. Eiffert, Banks W. Kooken, Melissa B. Miller, Luther A. Bartelt, Alan C. Kinlaw, Emily J. Ciccone

**Affiliations:** 1 Division of Infectious Diseases, Department of Medicine, University of North Carolina School of Medicine, Chapel Hill, NC, USA; 2 Carolina Antimicrobial Stewardship Program, University of North Carolina Medical Center, Chapel Hill, NC, USA; 3 Department of Epidemiology, University of North Carolina Gillings School of Global Public Health, Chapel Hill, NC, USA; 4 Division of Pharmaceutical Outcomes and Policy, University of North Carolina Eshelman School of Pharmacy, Chapel Hill, NC, USA; 5 University of North Carolina School of Medicine, Chapel Hill, NC, USA; 6 Department of Pathology and Laboratory Medicine, University of North Carolina, Chapel Hill, NC, USA; 7 Department of Microbiology and Immunology, University of North Carolina School of Medicine, Chapel Hill, NC, USA; 8 Center for Gastrointestinal Biology and Disease, Department of Medicine, University of North Carolina School of Medicine, Chapel Hill, NC, USA; 9 Cecil G. Sheps Center for Health Services Research, University of North Carolina, Chapel Hill, NC, USA

## Abstract

**Objective:**

To determine the frequency of antimicrobial management changes in response to positive gastrointestinal pathogen multiplex panel (GIP) results in patients with suspected infectious diarrhea, identify predictors of those changes, and assess their guideline adherence.

**Design:**

Single-center, retrospective cohort study

**Setting:**

Tertiary referral center, including ambulatory and acute care settings.

**Patients:**

Adult and pediatric patients with diarrhea and positive GIP evaluated in emergency department, inpatient, or outpatient settings between January 1 and December 31, 2018, were included. Patients considered immunocompromised due to underlying conditions and/or current/recent immunosuppressive therapies were excluded.

**Methods:**

The primary outcome of interest was any change in antimicrobial treatment in response to a positive GIP. The secondary outcome was adherence to pathogen-specific guideline-recommended management. Marginal standardization with logistic regression models was also used to assess predictors of antimicrobial management change.

**Results:**

The analysis included 193 patients with diarrhea and a positive GIP. The most frequently detected pathogens were norovirus, *Salmonella*, and *Campylobacter*. The median time from test collection to result was 31 hours [Q1, Q3: 24, 52]. Of the 193 patients, 71 (37%) experienced an antimicrobial management change, 61 of whom (86%) were considered guideline-adherent. In adjusted models, empiric antimicrobial treatment and testing in outpatient settings were significantly associated with experiencing an antimicrobial change.

**Conclusions:**

Around one-third of patients with a positive GIP test experienced a change in antimicrobials, and most of these changes were guideline-adherent. Most GIP tests ordered during the study period were negative, and most positive tests did not require antimicrobial therapy.

## Introduction

Acute diarrheal infection (ADI) is a major cause of outpatient visits and hospitalizations in the United States and among those traveling abroad, with an estimated incidence in the United States of 0.6 cases per person per year.^2,3^ Identifying the cause of ADI can be important for clinical management and treatment. Some pathogens require antimicrobials, while others are associated with worse outcomes when treated with antimicrobials (eg, O157:H7 Shiga-toxin producing *Escherichia coli*). Specific pathogens require mandatory reporting to implement public health control measures to reduce spread.^4^ Antimicrobial resistance varies among different enteropathogens, so the Infectious Disease Society of America (IDSA) guidelines are therefore increasingly pathogen-specific.^5,6^

Conventional stool pathogen diagnostics are time and labor-intensive, and overlapping clinical presentations across pathogens have led to the preference for multiplex molecular panels for evaluation of infectious diarrhea.^7^ GastrointestinaI pathogen multiplex panels (GIPs) detect multiple pathogens simultaneously and offer a rapid turnaround time and improved sensitivity compared to conventional tests.^8^ Rapid positive or negative results may decrease the time to optimal therapy. Studies suggest that use of GIPs may significantly lower overall antibiotic usage compared with conventional stool testing and may reduce hospitalization duration, healthcare costs, and unnecessary procedures such as colonoscopies.^9–14^ However, multiplex panels vary in cost depending on the number of pathogens detected, and their true cost-effectiveness is uncertain.^10,15–18^ Some studies suggest that use of GIPs may be associated with no change in prescribing practices or even increased antibiotic usage.^19–21^ Finally, there are limited data exploring clinical decision making and antibiotic management in response to GIP; in one study, 34% of antibiotics prescribed after positive GIP were considered “inappropriate.”^22^

More data are needed to evaluate the impact of multiplex GIPs on antimicrobial prescription practices, clinical outcomes, and healthcare costs. In this analysis, we aimed to quantify management changes after positive GIP result for the treatment of suspected ADI, determine the IDSA guideline-adherence of the antimicrobial management changes, and identify predictors of management changes made to identify clinical scenarios in which GIPs are most impactful. Such data could inform diagnostic and antimicrobial stewardship initiatives for cases of suspected infectious diarrhea.

## Methods

### Study design and setting

We conducted a retrospective study of patients with GIP testing for evaluation of diarrhea in inpatient, outpatient, or emergency room settings at any University of North Carolina Health facility from January 1 to December 31, 2018. Patients experiencing diarrhea at the time of GIP and testing positive for at least one pathogen were included. Individuals who tested positive for *Clostridioides difficile* by a separate assay but did not have a GIP performed were excluded. Immunocompromised patients were excluded from this study as clinical assessment and treatment of infectious diarrhea may differ in this population. Immunocompromised status was defined as having any of the following: solid organ or stem cell transplant, cancer with active chemotherapy within the past 6 months, neutropenia (absolute neutrophil count <500 cells/μL), human immunodeficiency virus infection with absolute CD4 count <200 cells/µL, primary immunodeficiency, or immunosuppressive medications including biologics and steroids with a prednisone equivalent dose of ≥20 mg/day. Patients for whom there was no documentation beyond the GIP result were also excluded.

### Study procedures

Study data were abstracted from electronic health records (EHRs) and managed using the UNC-hosted Research Electronic Data Capture (REDCap) tool.^23,24^ Data were collected on patient demographics, comorbidities (including diabetes mellitus, inflammatory bowel disease, irritable bowel syndrome [IBS], sickle cell disease, and cancer), site of sample collection, antibiotic use 30 days prior to testing, clinical presentation, time from sample collection to result, empiric usage of antibiotics or other testing (including invasive studies such as colonoscopy, endoscopy, and radiographic scans), GIP result, and any postGIP result clinical management changes including change in antibiotics prescribed, additional testing, avoidance of further testing, and initiation of isolation precautions.

### GIP testing

Gastrointestinal pathogen testing was performed using the Luminex xTAG Gastrointestinal Panel (Luminex Molecular Diagnostics, Austin, TX; see Supplemental Material for complete target list). Testing was performed per manufacturer’s instructions, and during the study period, GIP tests were batched by the laboratory once daily. Results for *C. difficile,* adenovirus 40/41, *Vibrio cholerae,* and *Entamoeba histolytica* were not reported in the EHR. *Clostridioides difficile* results were masked because of the PCR-based nature of the test, concern for detection of colonization, and availability of a separate in-house assay for *C. difficile.* For patients with a GIP order, the lab staff verified that patients did not have a positive *C. difficile* result from the same stool sample; if present, the ordering provider was asked to cancel the GIP. Ordering providers can request a GIP test be processed regardless, though it was rare. *C. difficile* results from the separate assay were recorded in our data set. *Vibrio cholerae* and *E. histolytica* results were masked because of the very low prevalence in our patient population and associated risk of false positives, and adenovirus 40/41 results were masked because a separate standalone adenovirus PCR test covering all serotypes was available.

### Definitions and outcomes

We defined diarrhea broadly as any diarrhea documented in the electronic medical record at the time of GIP order. The primary outcome of interest was any change in antimicrobial treatment in response to GIP result, referred to as antimicrobial management change. This included any initiation, discontinuation, or change in antimicrobial treatment that (a) occurred within 24 hours of the GIP test result or (b) was documented in electronic medical record notes as being in response to the GIP result. If an empirically prescribed antimicrobial treatment course was completed prior to GIP test result, this was not included as a management change. Secondary outcomes included guideline adherence based on the 2017 IDSA guidance document for infectious diarrhea and predictors of antimicrobial management change.^6^ A rubric was used to assign guideline adherence of antimicrobial management when there was ambiguity or lack of explicit guidelines for the pathogen (Table S1). We defined potential predictors of antimicrobial management change as sex, race and ethnicity, age, insurance status, comorbidities, empiric use of antimicrobials, antibiotic use 30 days prior to testing, vomiting at time of GIP test, and test collection site (Figure S1).

### Data analysis

Descriptive statistics were used to report the study population’s demographic and clinical characteristics and GIP test results. We created a Sankey diagram to illustrate the impact of a positive GIP result on antimicrobial management changes, stratified by empiric antibiotic use.^25^ To assess associations between predictors and the outcome of antimicrobial management change, we estimated crude and adjusted risk differences for each predictor, using logistic regression models that adjusted for confounding using marginal standardization.^26^ We obtained 95% confidence intervals using bootstrap processes with 2000 resamples. To identify relevant adjustment sets to handle confounding for each predictor, we constructed a directed acyclic graph describing the relationships between each variable in our data (see example in Figure S1).^27,28^ Analyses were completed using SAS version 9.4 (SAS Institute, Inc. Cary, NC) and R Statistical Software version 4.4.1 (The R Foundation, Vienna, Austria).

## Results

Of 2,333 GIP tests performed during the study period, there were 241 (10.3%) patients with positive results. Of these, there were 46 positives from immunocompromised patients excluded, and 2 patients were excluded due to lack of data. Ultimately, 193 patients with diarrhea and a positive GIP were included in the analysis. Of these, 55% were male, and the median age was 31 years (interquartile range [IQR] 5, 56) (Table 1). Presenting symptoms and initial antimicrobial management are described in Table S2.

**Table 1.** Baseline demographics of cohort with acute diarrheal illness

**Category**	**N=193**
Male, n (%)	106 (55)
Race, n (%)	
White, non-Hispanic	125 (65)
Black, non-Hispanic	30 (16)
Hispanic	21 (11)
Asian	7 (4)
Other, unknown, or not documented	10 (5)
Age (years), median [IQR]	31 [5, 56]
Age group (years), n (%)	
0–5.9	51 (26)
6–17.9	15 (8)
18–34.9	38 (20)
35–64.9	65 (34)
≥65	24 (12)
Insurance coverage, n (%)	
Private	112 (58)
Medicare/Medicaid	64 (33)
Uninsured	17 (9)
Smoking history, n (%)	
Never	129 (67)
Former	39 (20)
Current	25 (13)
Comorbidities, n (%)	
At least one of the following:	42 (22)
Cancer	19 (10)
Diabetes	17 (9)
Irritable bowel syndrome	8 (4)
Inflammatory bowel disease	3 (2)

Note. IQR, interquartile range.

Outpatient clinics performed 73 of 193 (38%) GIP (Table 2). Among patients tested in the emergency department (ED), 21 of 65 (32%) were subsequently admitted. The median time from GIP collection to test result was 31 hours [IQR 24, 52] and did not differ between those with and without an antimicrobial change (Table 2). In 76 hospitalizations, 28 (37%) positive GIP results were reported after discharge. The most frequently identified pathogens were norovirus (n = 55), *Salmonella* (n = 44), and *Campylobacter* (n = 35). Nine individuals tested positive for more than one pathogen, of which 2 were positive for *C. difficile* (1 co-infected with *Salmonella* and 1 with *E. coli*).

**Table 2.** Details of gastrointestinal pathogen testing

	Total (n=193)	Antimicrobial change (n=71)	No antimicrobial change (n=122)
Testing site, n (%)			
ED	65 (34)	19 (27)	46 (38)
Outpatient clinic	73 (38)	33 (47)	40 (33)
Inpatient (floor, stepdown, or ICU)	55 (29)	19 (27)	36 (30)
Antibiotics in 30 d before test, n (%)			
Yes	37 (19)	17 (24)	20 (16)
No	150 (78)	53 (75)	97 (80)
Unknown	6 (3)	1 (1)	5 (4)
Time from collection to result (hours), median [IQR]	31 [24, 52]	30 [21, 50]	31 [25, 56]
Empiric antibiotics received, n (%)	56 (29)	27 (38)	29 (24)
Pathogen detected by GIP test, n (%)			
* Campylobacter*	35 (18)	17 (24)	18 (15)
* Salmonella*	44 (23)	24 (34)	20 (16)
* E. coli* ^a^	18 (9)	9 (13)	9 (7)
Norovirus	55 (29)	6 (9)	49 (40)
Other^b^	32 (17)	11 (15)	21 (17)
Multiple pathogens	9 (5)	4 (6)	5 (4)

Note. GIP, gastrointestinal pathogen panel; ICU, intensive care unit; IQR, interquartile range.

a Includes *E. coli* 0157, Enterotoxigenic *E. coli,* and Shiga-toxin producing *E. coli.*

b Other pathogens: *Shigella*, *Giardia*, *Cryptosporidium*, and Rotavirus.

A total of 73 of 193 patients (38%) experienced at least one management change in response to a positive GIP, with 86 changes observed (Table 3). Antimicrobial management changes made up 71 of 86 (83%) of all identified clinical management changes, and most of these were initiation of new antimicrobials (66%, 47 of 71). No antimicrobial changes occurred in patients tested in the intensive care unit (n = 3). There were 15 non-antimicrobial management changes, which included Department of Health reporting, letters provided (daycare, work), cessation of other medication (laxatives, steroids), and extension of antibiotic course (without change in antimicrobial).

**Table 3.** Changes in clinical management after positive GIP result (n=86)^*^

Antimicrobial management changes, n (%)	
Antimicrobial changed	8 (4)
Antimicrobial discontinued	16 (8)
Antimicrobial started	47 (24)
Other management changes, n (%)	
Isolation precaution changed	6 (3)
Other procedure or testing performed	3 (2)
Other procedure or testing avoided	6 (3)
Other^a^	13 (7)

Note. GIP, gastrointestinal pathogen panel.

* There were 86 observed changes for 73 individuals.

a Includes: Reported to the Department of Health, counseling provided (eg, daycare, work), other medication stopped (eg, laxatives, steroids), antibiotic course extended but not changed.

**Figure 1.**
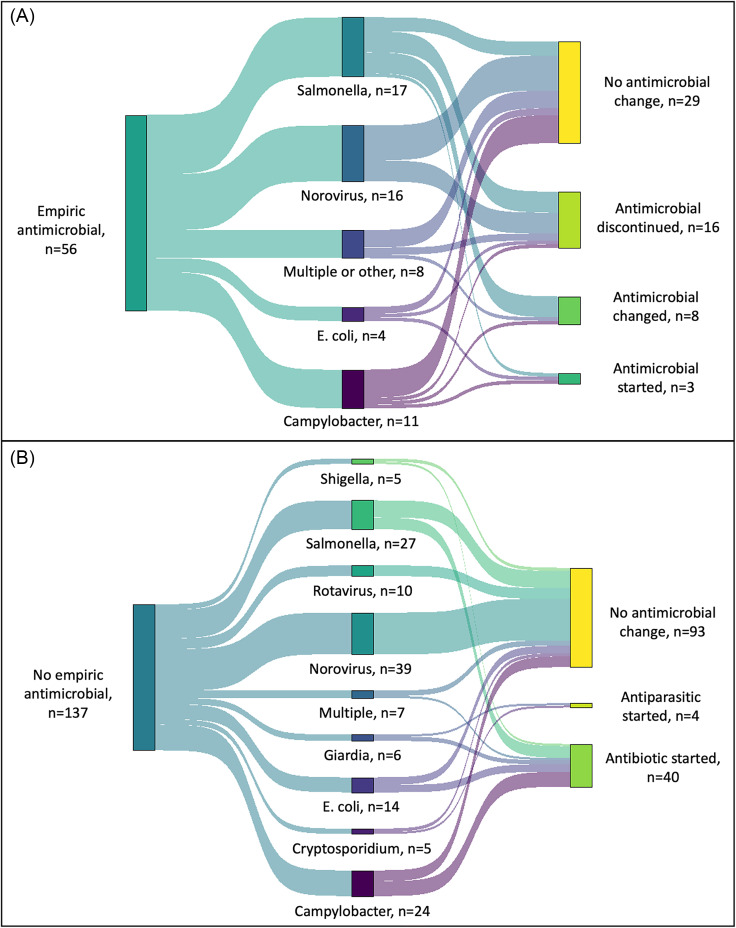
Sankey diagrams demonstrating the impact of GIP result on antimicrobial use by pathogen detected in those who received empiric antimicrobial treatment (**A**) prior to test result and those who did not (**B**). “*E. coli”* includes Shiga-toxin producing *Escherichia coli*, enterotoxigenic *E. coli*, and *E. coli* O157:H7.

Overall, 56 of 193 patients (29%) were prescribed empiric antimicrobials, most of whom were adults aged 18 years and older (84%, 47 of 56) (Table 2). Empiric antimicrobials were discontinued in 16 of 56 patients (29%) in response to a positive GIP result. In the inpatient setting, more patients were treated with empiric antibiotics (46%, 25 of 55) compared with patients tested in outpatient clinics (21%, 15 of 73) and ED settings (25%,16 of 65), and subsequently fewer inpatients experienced antimicrobial initiation after positive GIP result (11%, 6 of 55) compared with outpatient clinics (37%, 27 of 73) and the ED (22%, 14 of 65). Fewer antimicrobial changes were made after GIP in children under 18 years old (23%, 15 of 66) compared with adults aged 18 years and older (44%, 56 of 127). Antibiotics were most often discontinued for norovirus and started for *Campylobacter* (Figure 1). In 2 patients positive for *C. difficile* by the separate assay, both were treated for *C. difficile* infection only, and the organism detected on GIP was considered colonization.

**Figure 2.**
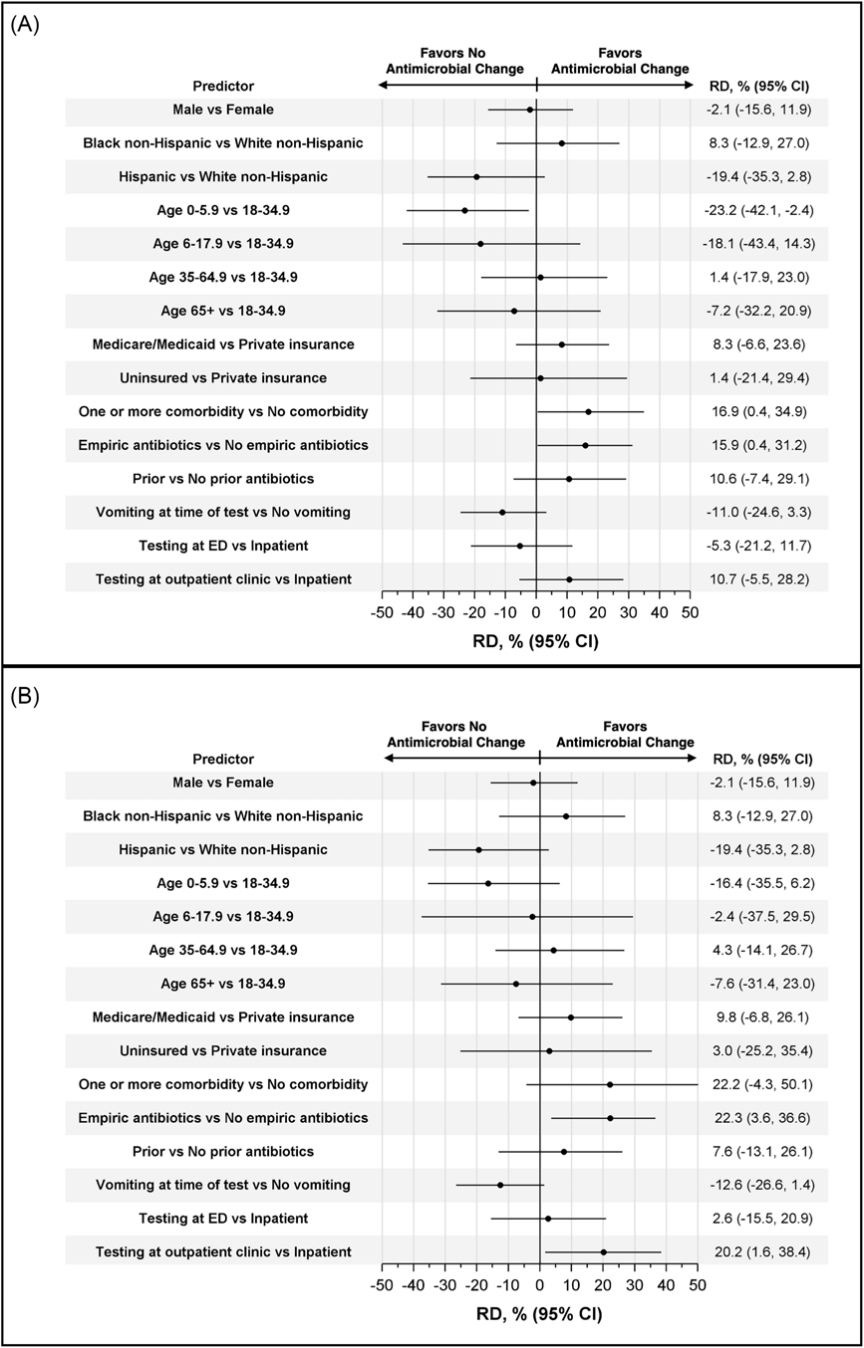
Forest plots detailing predictors of antimicrobial management change. (A) Displays risk differences and 95% CI for the crude results. (B) Displays risk differences and 95% CI for adjusted results. For both crude and adjusted models, there were insufficient counts of patients with documented “Asian” or “other” race/ethnicity; no estimates for those groups were made.

In unadjusted regression models, the age range of 0–5.9 years was associated with a 23% lower risk of experiencing an antimicrobial change after GIP result using age 18–34.9 years as the referent (risk difference [RD] 95% CI: −42%, −2%), whereas having one or more comorbidities (RD 22%, 95% CI: 4%, 50%) and initial treatment with antimicrobials (RD 16%, 95% CI: 0%, 35%) were associated with an increased risk of experiencing an antimicrobial change (Figure 2). In adjusted models, receiving empiric antimicrobial treatment (RD 22%, 95% CI: 4%, 37%) and testing performed in outpatient settings (RD 20%, 95% CI: 2%, 38%) were significantly associated with experiencing an antimicrobial change in response to a positive GIP test. For both unadjusted and adjusted models, there were no significant associations between other covariates and the primary outcome of interest.

Most antimicrobial management changes (86%, 61 of 71) were adherent to the IDSA infectious diarrhea guidelines; 16 of these changes were antimicrobial discontinuation. Guideline adherence was similar among patients who received empiric antibiotics (89%) and those who did not (84%). More antimicrobial management changes observed were guideline-adherent in children younger than 18 years old (93%, 14 of 15) compared with adults aged 18 years and older (84%, 47 of 56). Of 10 guideline non-adherent antimicrobial changes, one was in a pediatric patient (antibiotic started), and 9 were in adults (n = 8 antibiotics started, n = 1 antibiotics discontinued). The majority were treated in the outpatient setting (n = 7) or ED (n = 2). Ultimately, 9 of 47 (19%) antimicrobials initiated in response to GIP were non-adherent to guidelines (5% of the full cohort). The organism most often treated in guideline non-adherent cases was enterotoxigenic *E. coli* (9 of 10).

## Discussion

There are limited data evaluating clinical decision-making and antibiotic management in response to GIP, especially in adults.^9,13,19,22^ In this analysis, most GIP tests during the study period resulted negative or were positive for pathogens or sent in clinical contexts in which the IDSA does not recommend antimicrobial treatment. Approximately one-third of positive GIP tests led to a change in antimicrobial therapy, which was most commonly initiation of a new antimicrobial. At our institution, testing performed in outpatient settings as well as the receipt of empiric antimicrobials prior to test result were most strongly associated with an antimicrobial management change. Most antimicrobial changes were adherent to existing pathogen-specific IDSA guidelines. However, 20% of antibiotics initiated in response to GIP were considered non-adherent to guidelines, and most of these non-adherent changes occurred in adults in the outpatient setting.

Most existing studies examining the clinical impact of GIP have been conducted in pediatric populations, reporting metrics such as the proportion of patients who experienced an antibiotic management change (12–50%), which is consistent with our findings in adults and children.^8,29–31^ This study is one of the very few attempting to assess antibiotic prescribing practices after GIP, the largest study to do so, and the only using published expert guidance for infectious diarrhea to assess appropriateness.^19,21,22^ Most of the antimicrobial changes observed in response to GIP were adherent to the reference guideline, though we did not assess the appropriateness of antimicrobial treatments that were completed before or unchanged after GIP result, nor did we have sufficient patient-specific data to account for other co-occurring conditions that may have influenced antibiotic changes. Notably, one prior study determined that 34% of antimicrobial use was inappropriate based on the detected pathogen; that study included only adult patients. In our study, adult patients experienced the bulk of guideline-discordant antimicrobial changes after GIP.^22^

The most frequently identified pathogens were similar to those previously reported.^9,13^ In our study, a very small number (n = 2) were also positive for *C. difficile* by a separate GDH/toxin with reflex to PCR assay, but this reflects a laboratory policy to request cancellation of GIP orders for patients with a separate positive *C. difficile* test on the same stool sample. The median time to GIP result at our institution (31 h) was slightly longer than previously published median times to GIP result.^12,14^ Our study included patients who were seen in outpatient clinics who tend to be less acutely ill, while existing literature has thus far predominantly included sicker patients presenting at the ED and/or patients from an inpatient setting. Additionally, GIP testing was batch run during the study period, whereas other studies assessed random-access multiplex platforms; these factors likely contributed to our longer time to result.^12–14^ Importantly, we found that among inpatients or patients who were admitted to the hospital from the ED, 37% of GIP tests resulted after the patient had been discharged. We hypothesize that prolonged turnaround time during the analysis period for this batched multiplex test impacted the ability for providers to make management changes after GIP result, as short antimicrobial courses could have been completed before GIP results became available. It is possible that shortening time to result either through immediate and/or using a more rapid platform could increase likelihood of a resulting management change.

A strength of our study lies in the granularity of data collected, including exact timing of testing and management changes, antimicrobial treatment(s), and types of management change documented through extensive chart adjudication. Its limitations are in part due to the retrospective nature of this study; we may be missing management changes not clearly documented in the EHR. In addition, the etiology of diarrhea may include pathogens not captured on the GIP, and it is possible that some results may represent prolonged shedding from a previous infection with the current case of diarrhea caused by a non-infectious etiology such as laxative use or postinfectious IBS. Our lab refers antibiotic susceptibility testing (AST) on intestinal pathogens to state health laboratories when indicated. The increasing prevalence of antimicrobial resistance, for example, in *Campylobacter* and *Salmonella*, is an important consideration. AST data are frequently missing for enteropathogens and could affect the accuracy of guideline concordance. Additionally, we excluded certain patient populations, including severely immunocompromised hosts, and cannot comment on prescribing practices after GIP for this population. Finally, these data are from 2018; as of October 2024, our institution’s GIP turnaround time is now faster than the test used during the study period, and stewardship practices and diagnostics have evolved, which may limit study generalizability. Implementation of a rapid GIP may facilitate faster utilization of results for treatment decision-making, prior to hospital or ED discharge or completion of empiric antimicrobials.

In conclusion, our results suggest that a focus on interpretation of GIP results, specifically in adults, may improve diagnostic and prescribing practices for ADI. Decision-making after results may be affected by empiric treatment decisions, turnaround times, ordering department, plan for follow-up, and heterogeneity in interpretation of existing guidance documents for infectious diarrhea. Updated pathogen- or panel-specific guidelines for the interpretation of results and treatment of diarrhea would be useful to provide clarity to clinicians, improve pathogen-specific treatment homogeneity, and reduce unnecessary antimicrobial use. As most intestinal pathogens are state-reportable infections, an informed clinical guideline could optimize the use of GIP for both clinical providers and public health surveillance. In addition, further study of the clinical contexts in which non-infectious causes of diarrhea are most likely, and subsequent development of clinical decision support tools, could improve diagnostic stewardship for these tests.^32,33^

## Supporting information

10.1017/ash.2025.10184.sm001 Mackow et al. supplementary material Mackow et al. supplementary material

## Supplementary material

The supplementary material for this article can be found at https://doi.org/10.1017/ash.2025.10184

## Research transparency and reproducibility

Access to deidentified data and code can be made available upon request and appropriate ethics approvals.

## References

[1] Mackow NA , Eiffert SR , Kinlaw AC , Kooken BW , Miller MB , Bartelt LA , Ciccone EJ . Clinical management changes after positive multiplex gastrointestinal pathogen panel testing for evaluation of diarrhea. Open Forum Infect Dis 2001;9:ofac492.1626.

[2] Riddle MS , DuPont HL , Connor BA. ACG clinical guideline: diagnosis, treatment, and prevention of acute diarrheal infections in adults. Am J Gastroenterol 2016;111:602–622. 27068718 10.1038/ajg.2016.126

[3] Scallan E , Hoekstra RM , Angulo FJ , et al. Foodborne illness acquired in the United States--major pathogens. Emerg Infect Dis 2011;17:7–15. 21192848 10.3201/eid1701.P11101 PMC3375761

[4] Wikswo ME , Roberts V , Marsh Z , et al. Enteric illness outbreaks reported through the national outbreak reporting system-united States, 2009-2019. Clin Infect Dis 2022;74:1906–1913. 34498027 10.1093/cid/ciab771 PMC11194694

[5] Hatchette TF , Farina D. Infectious diarrhea: when to test and when to treat. Cmaj 2011;183:339–344. 21173060 10.1503/cmaj.091495 PMC3042443

[6] Shane AL , Mody RK , Crump JA , et al. Infectious Diseases Society of America clinical practice guidelines for the diagnosis and management of infectious diarrhea. Clin Infect Dis 2017; 65:e45–e80. 29053792 10.1093/cid/cix669 PMC5850553

[7] Binnicker MJ. Multiplex molecular panels for diagnosis of gastrointestinal infection: performance, result interpretation, and cost-effectiveness. J Clin Microbiol 2015;53:3723–3728. 26311866 10.1128/JCM.02103-15 PMC4652086

[8] Cotter JM , Thomas J , Birkholz M , Ambroggio L , Holstein J , Dominguez SR. Clinical impact of a diagnostic gastrointestinal panel in children. Pediatrics 2021;147:e2020036954. PMID: 33837134. 10.1542/peds.2020-036954 33837134

[9] Axelrad JE , Freedberg DE , Whittier S , Greendyke W , Lebwohl B , Green DA. Impact of gastrointestinal panel implementation on health care utilization and outcomes. J Clin Microbiol 2019;57:e01775-18. PMID: 30651393; PMCID: PMC6425162 10.1128/JCM.01775-18 PMC6425162 30651393

[10] Beal SG , Tremblay EE , Toffel S , Velez L , Rand KH. A gastrointestinal PCR panel improves clinical management and lowers health care costs. J Clin Microbiol 2017;56:e01457-17. PMID: 29093106; PMCID: PMC5744222 10.1128/JCM.01457-17 PMC5744222 29093106

[11] Machiels JD , Cremers AJH , van Bergen-Verkuyten M , et al. Impact of the BioFire FilmArray gastrointestinal panel on patient care and infection control. PLoS One 2020;15:e0228596. 32027698 10.1371/journal.pone.0228596 PMC7004333

[12] Sobczyk J , Jain S , Sun X , et al. Comparison of multiplex gastrointestinal pathogen panel and conventional stool testing for evaluation of patients with HIV infection. Open Forum Infect Dis 2020;7:ofz547. 31976355 10.1093/ofid/ofz547 PMC6970129

[13] Torres-Miranda D , Akselrod H , Karsner R , et al. Use of BioFire FilmArray gastrointestinal PCR panel associated with reductions in antibiotic use, time to optimal antibiotics, and length of stay. BMC Gastroenterology 2020;20:246. 32727381 10.1186/s12876-020-01394-w PMC7392718

[14] Yoo IH , Kang HM , Suh W , et al. Quality improvements in management of children with acute diarrhea using a multiplex-PCR-based gastrointestinal pathogen panel. Diagnostics (Basel) 2021;11. 10.3390/diagnostics11071175 PMC8303787 34203426

[15] Freeman K , Mistry H , Tsertsvadze A , et al. Multiplex tests to identify gastrointestinal bacteria, viruses and parasites in people with suspected infectious gastroenteritis: a systematic review and economic analysis. Health Technol Assess 2017;21:1–188. 10.3310/hta21230 PMC5494512 28619124

[16] DiDiodato G , Allen A , Bradbury N , et al. The efficacy of the BioFire FilmArray gastrointestinal panel to reduce hospital costs associated with contact isolation: a pragmatic randomized controlled trial. Cureus 2022;14:e27931. 36120274 10.7759/cureus.27931 PMC9464456

[17] Goldenberg SD , Bacelar M , Brazier P , Bisnauthsing K , Edgeworth JD. A cost benefit analysis of the Luminex xTAG gastrointestinal pathogen panel for detection of infectious gastroenteritis in hospitalised patients. J Infect 2015;70:504–511. 25449904 10.1016/j.jinf.2014.11.009

[18] Moon RC , Bleak TC , Rosenthal NA , et al. Relationship between diagnostic method and pathogen detection, healthcare resource use, and cost in U.S Adult outpatients treated for acute infectious gastroenteritis. J Clin Microbiol 2023;61:e0162822. 36645308 10.1128/jcm.01628-22 PMC9945572

[19] Meltzer AC , Newton S , Lange J , et al. A randomized control trial of a multiplex gastrointestinal PCR panel versus usual testing to assess antibiotics use for patients with infectious diarrhea in the emergency department. J Am Coll Emerg Physicians Open 2022;3:e12616. 35072157 10.1002/emp2.12616 PMC8760946

[20] O’Neal N , Murray H , Dash S , Al-Hasan MN , Justo JA , Bookstaver PB. Evaluating appropriateness and diagnostic stewardship opportunities of multiplex polymerase chain reaction gastrointestinal testing within a hospital system. Ther adv infect dis 2020;7:2049936120959561. 33014363 10.1177/2049936120959561 PMC7513010

[21] Saling CF , Seville MT , Patron RL. The effect of gastrointestinal pathogen panel (GIP) on antibiotic management. Antimicrob Steward Healthc Epidemiol 2021;1:e5. 36168483 10.1017/ash.2021.162 PMC9495426

[22] Cetin S , Telli E , Sahin AM , et al. Gastrointestinal PCR panel results and antibiotic use in acute gastroenteritis cases: how appropriate are we in our usage? Indian J Med Microbiol 2024;47:100536. 38316393 10.1016/j.ijmmb.2024.100536

[23] Harris PA , Taylor R , Minor BL , et al. The REDCap consortium: building an international community of software platform partners. J Biomed Inform 2019;95:103208. 31078660 10.1016/j.jbi.2019.103208 PMC7254481

[24] Harris PA , Taylor R , Thielke R , Payne J , Gonzalez N , Conde JG. Research electronic data capture (REDCap)--a metadata-driven methodology and workflow process for providing translational research informatics support. J Biomed Inform 2009;42:377–381. 18929686 10.1016/j.jbi.2008.08.010 PMC2700030

[25] Otto E , Culakova E , Meng S , et al. Overview of Sankey flow diagrams: focusing on symptom trajectories in older adults with advanced cancer. J Geriatr Oncol 2022;13:742–746. 35000890 10.1016/j.jgo.2021.12.017 PMC9232856

[26] Naimi AI , Whitcomb BW. Estimating risk ratios and risk differences using regression. Am J Epidemiol 2020;189:508–510. 32219364 10.1093/aje/kwaa044

[27] Greenland S. Quantifying biases in causal models: classical confounding vs collider-stratification bias. Epidemiology 2003;14:300–306. 12859030

[28] Greenland S , Pearl J , Robins JM. Causal diagrams for epidemiologic research. Epidemiology 1999;10:37–48. 9888278

[29] Ergun D , Kacar P , Ozbakir H , et al. The impact of multiplex nested gastrointestinal PCR panel in children with gastroenteritis requiring pediatric infectious disease consultation. Eur J Pediatr 2024;184:85. 39680183 10.1007/s00431-024-05918-4

[30] Sever A , Ben Zvi H , Melamed SB , Sachs N , Krause I , Bilavsky E. Clinical impact of biofire gastrointestinal panel testing for hospitalised children with acute gastroenteritis. Acta Paediatr 2023;112:505–509. 36447381 10.1111/apa.16610

[31] Truong J , Cointe A , Le Roux E , et al. Clinical impact of a gastrointestinal PCR panel in children with infectious diarrhoea. Arch Dis Child 2022;107:601–605. 34921002 10.1136/archdischild-2021-322465

[32] Marcelin JR , Brewer C , Beachy M , et al. Hardwiring diagnostic stewardship using electronic ordering restrictions for gastrointestinal pathogen testing. Infect Control Hosp Epidemiol 2019;40:668–673. 31012405 10.1017/ice.2019.78

[33] Saif NT , Dooley C , Baghdadi JD , Morgan DJ , Coffey KC. Clinical decision support for gastrointestinal panel testing. Antimicrob Steward Healthc Epidemiol 2024;4:e22. 38415090 10.1017/ash.2024.15 PMC10897720

