# Supplementary material for: Antimicrobial changes made for suspected infectious diarrhea after positive gastrointestinal pathogen panel result
Source: Antimicrob Steward Healthc Epidemiol. 2025 Oct 20;5(1):e281. doi: 10.1017/ash.2025.10184 (PMC12538352; doi:10.1017/ash.2025.10184)

**Supplemental Material**

**Luminex xTAG Gastrointestinal Panel Full Target List**

*Campylobacter*

*Adenovirus 40/41*

*Cryptosporidium*

*Clostridium difficile, Toxin A/B**

*Norovirus GI/GII*

*Entamoeba histolytica**

*Escherichia coli O157*

*Rotavirus A*

*Giardia*

*Enterotoxigenic E. coli (ETEC) LT/ST*

*Shiga-likeToxin producing E. coli (STEC) stx1/stx2*

*Salmonella*

*Shigella*

*Vibrio cholerae**

*Results masked in electronic health record per laboratory protocols

| **Table S1**. Rubric for Assessing guideline-adherence of Antibiotic use for diarrheal illness after positive GIP based on 2017 IDSA guidance document for Infectious Diarrhea | | | |
| --- | --- | --- | --- |
| **Pathogen** | **Recommend antibiotics/antimicrobials?** | **Appropriate Antibiotics** | **Notes** |
| *Salmonella spp.* | Yes, if:   - ≤ 3 months old - > 50 years old with suspected atherosclerosis - Immunocompromised - Cardiac disease (valvular or endovascular) - Known significant joint disease | Ceftriaxone; ciprofloxacin, ampicillin, TMP-SMX, azithromycin, amoxicillin | Per IDSA Guidance document: *Consider* antibiotics for groups at increased risk of infection, but most infections do not need treatment if due to *S. enterica.*  The Luminex xTAG does not distinguish between *S. typhi* and *S. enterica* however most *Salmonella* enteritis cases in the US are due to *S. enterica.*  Any fluoroquinolone was considered ok if used in the right context. |
| *Shigella spp.* | Yes | Azithromycin, ciprofloxacin, or ceftriaxone for adult and pediatric infections; alternatively, TMP-SMX or ampicillin appropriate for adults. | Any fluoroquinolone was considered ok to use. |
| *Campylobacter spp.* | Yes | Azithromycin or ciprofloxacin | Any fluoroquinolone was considered ok.  Early antibiotic therapy may reduce duration of illness, but symptoms are self-limited, regardless.  While fluoroquinolone resistance can develop, antimicrobial therapy does not appear to prolong bacterial shedding.  Thus, the IDSA guidance concludes that the benefits of treating *Campylobacter* ADI are small but so are risks. It is reasonable to treat patients with severe or prolonged disease, or if immunocompromised with uncomplicated ADI. |
| *E. coli -* Shiga-toxin producing (STEC) | No | n/a | IDSA guidance document specifies that antibiotics should be avoided for STEC, Shiga-2, or STEC of unknown genotype. There is insufficient evidence to make a recommendation for patients with other STEC infections. |
| *E. coli* 0157 (EPEC) | No | n/a | IDSA guidance document does not comment on ETEC or EPEC; we scored adherence based on guidance for other STEC infections |
| *E. coli* (ETEC) | No | n/a | IDSA guidance document does not comment on ETEC or EPEC; we scored adherence based on guidance for other STEC infections |
| Cryptosporidium | Yes | Nitazoxanide. If HIV-infected, effective ART with or without nitazoxanide appropriate | IDSA guidance document does not comment on treating with supportive care alone for immunocompetent persons |
| Giardia | Yes | Tinidazole, Nitazoxanide, metronidazole (alternative) | Metronidazole not FDA approved for treatment of Giardiasis |
| Norovirus | No | n/a |  |
| Rotavirus | No | n/a |  |
| Abbreviations: Acute diarrheal illness (ADI), Antiretroviral therapy (ART), enterotoxigenic *E. coli* (ETEC), enteropathogenic *E. coli* (EPEC), Food and Drug Administration (FDA), Gastrointestinal pathogen panel (GIP), Human immunodeficiency virus (HIV), Shiga-Toxin producing *E. coli* (STEC), trimethoprim-sulfamethoxazole (TMP-SMX).  For each management change, based on the GIP result and available clinical data, changes were considered adherent or not based on recommendations as they were written in the 2017 Infectious Diseases Society of America (IDSA) guidance document. | | | |

| **Table S2.** Clinical presentation and initial evaluation and management of study population | | |
| --- | --- | --- |
| **Presenting Symptoms**, n (%) | |  |
| Abdominal pain/cramping | | 95 (49) |
| Vomiting | | 82 (43) |
| Fever^a^ | | 81 (42) |
| Abdominal tenderness | | 54 (28) |
| Blood in stool^b^ | | 52 (27) |
| **Initial Antimicrobial Treatment**, n (%) | | **56 (29)** |
|  | Ciprofloxacin | 24 (12) |
|  | Metronidazole | 21 (11) |
|  | Piperacillin/tazobactam | 12 (6) |
|  | Ceftriaxone | 7 (4) |
|  | Azithromycin | 7 (4) |
|  | Cefepime | 6 (3) |
|  | IV Vancomycin | 6 (3) |
|  | PO Vancomycin | 3 (2) |
|  | Levofloxacin | 3 (2) |
|  | Doxycycline | 3 (2) |
|  | Meropenem | 1 (1) |
|  | Other^c^ | 5 (3) |
| ^a^Documented or subjective fever  ^b^Melena or bright red blood  ^c^Other antimicrobials: Acyclovir, Gentamicin, Linezolid, Mebendazole, Tinidazole | | |

**Figure S1**: Directed acyclic graph showing associations between variables in the analytic dataset. Minimal sufficient adjustment sets varied for each predictor. Multivariable results of each predictor were adjusted as follows:

Sex: none

Race and ethnicity: none

Age: Race and ethnicity, Sex

Insurance status: Age, Race and ethnicity

Comorbidities: Age, insurance, Race and ethnicity

Empiric use of antimicrobials: Antibiotic use 30 days prior to testing, Age, Comorbidities, Insurance status, Race and ethnicity, Sex, vomiting at time of GIP test, Testing site

Antibiotic use 30 days prior to testing: Age, Comorbidities, Insurance status, Race and Ethnicity, Sex

Vomiting at time of GIP test: Antibiotic use 30 days prior to testing, Age, Comorbidities

Testing site: Antibiotic use 30 days prior to testing, Age, Comorbidities, Insurance status, Vomiting at time of GIP test


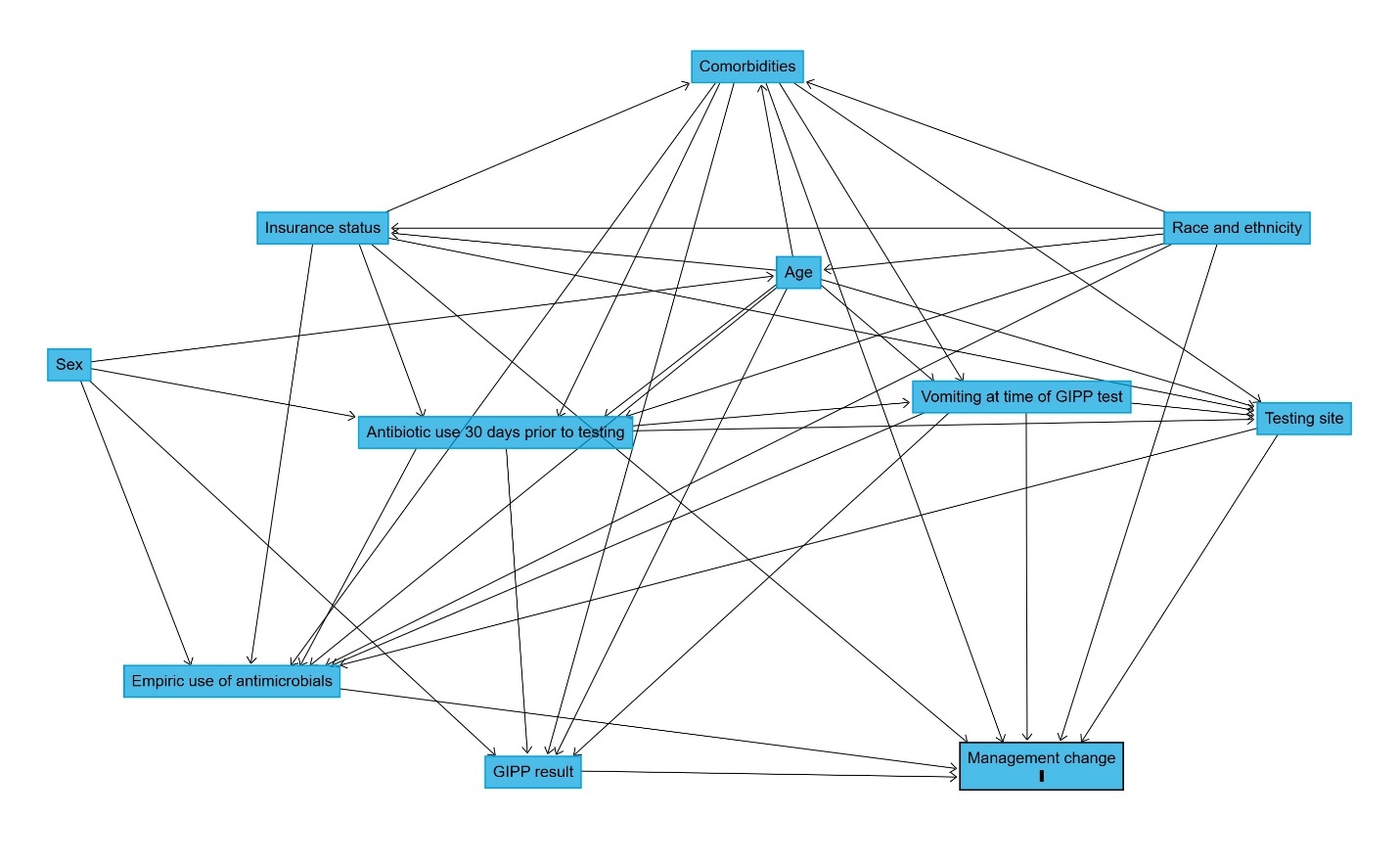

Supplement: Mackow et al. supplementary material [file S2732494X25101848sup001.docx]
